# The Epidural Ligaments (of Hofmann): A Comprehensive Review of the Literature

**DOI:** 10.7759/cureus.779

**Published:** 2016-09-13

**Authors:** Gabrielle G Tardieu, Christian Fisahn, Marios Loukas, Marc Moisi, Jens Chapman, Rod J Oskouian, R. Shane Tubbs

**Affiliations:** 1 Department of Anatomy, St. George’s University; 2 Orthopedic Surgery, Swedish Neuroscience Institute; 3 Department of Trauma Surgery, BG University Hospital Bergmannsheil, Bochum, Germany; 4 Department of Anatomy, St. George's University; 5 Seattle Science Foundation; 6 Neurological Surgery, Wayne State University; 7 Orthopedics Spine Surgery, Swedish Neuroscience Institute; 8 Neurosurgery, Complex Spine, Swedish Neuroscience Institute; 9 Neurosurgery, Seattle Science Foundation

**Keywords:** anatomy, spine, dura mater, extradural, epidural ligaments

## Abstract

The epidural space contains the internal vertebral venous plexus, adipose, and other connective tissues. In the anatomical literature, there are nonspecific descriptions of varying fibrous connective tissue bands in the epidural space, mainly mentioned in the lumbar region, that tether the dural sac to the posterior longitudinal ligament, the vertebral canal, and the ligamentum flavum. These ligaments have been termed as Hofmann’s ligaments. This review expands on the anatomy and function of Hofmann’s ligaments, increasing the awareness of their presence and serves as an impetus for further study of their histology, innervation, and function.

## Introduction and background

**Figure 1:**
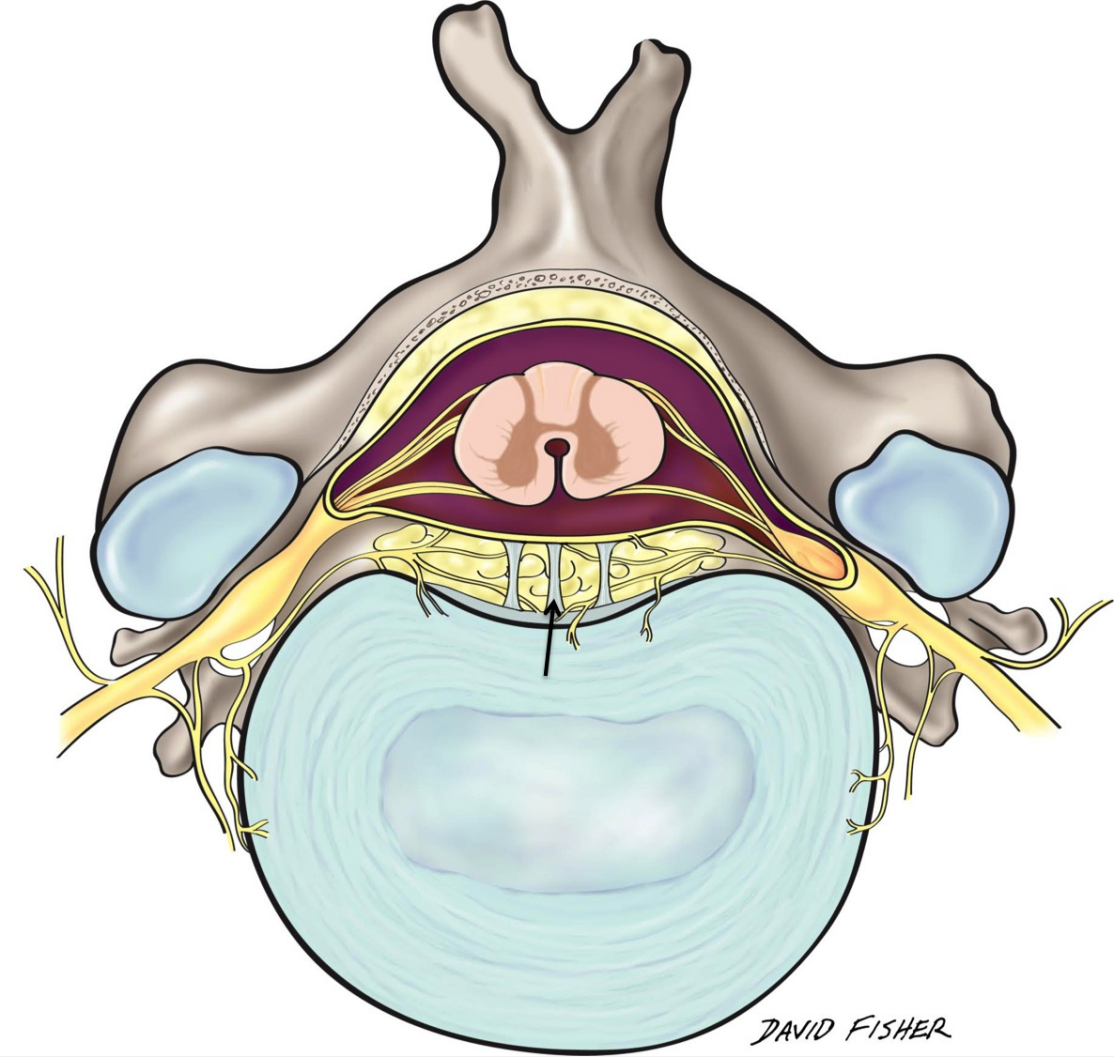
Schematic drawing illustrating Hofmann’s ligaments (arrow) in the epidural space.

Reports in the anatomic literature on the anterior spinal epidural space mention fibrous bands of connective tissue, which connect the anterior dural sac to the posterior longitudinal ligament (PLL) [1] as well as to the spinal canal. However, the anatomy and function of these bands (Hofmann’s ligaments) (Figure 1) are not well understood. Therefore, the objective of this review is to elaborate further and understand the anatomy and importance of these ligaments.

The epidural space is an important anatomical site for spine surgeons, anesthesiologists, anesthetists, and radiologists. Although controversial, the spinal epidural space is a true potential space that is located between the spinal dural sac and the bony vertebral canal [2-3]. It is lined by a thin layer of “mesenchymal epithelium” [4]. The anterior epidural space contains the internal vertebral venous plexus, connective tissue, and PLL. Husemeyer and White [5] stated that the anterior dura mater is firmly attached to the PLL, and the dural sac has no other attachments, bony or ligamentous, to the vertebral canal although, as seen below, others have found otherwise. 

## Review

### Anatomy

**Figure 2:**
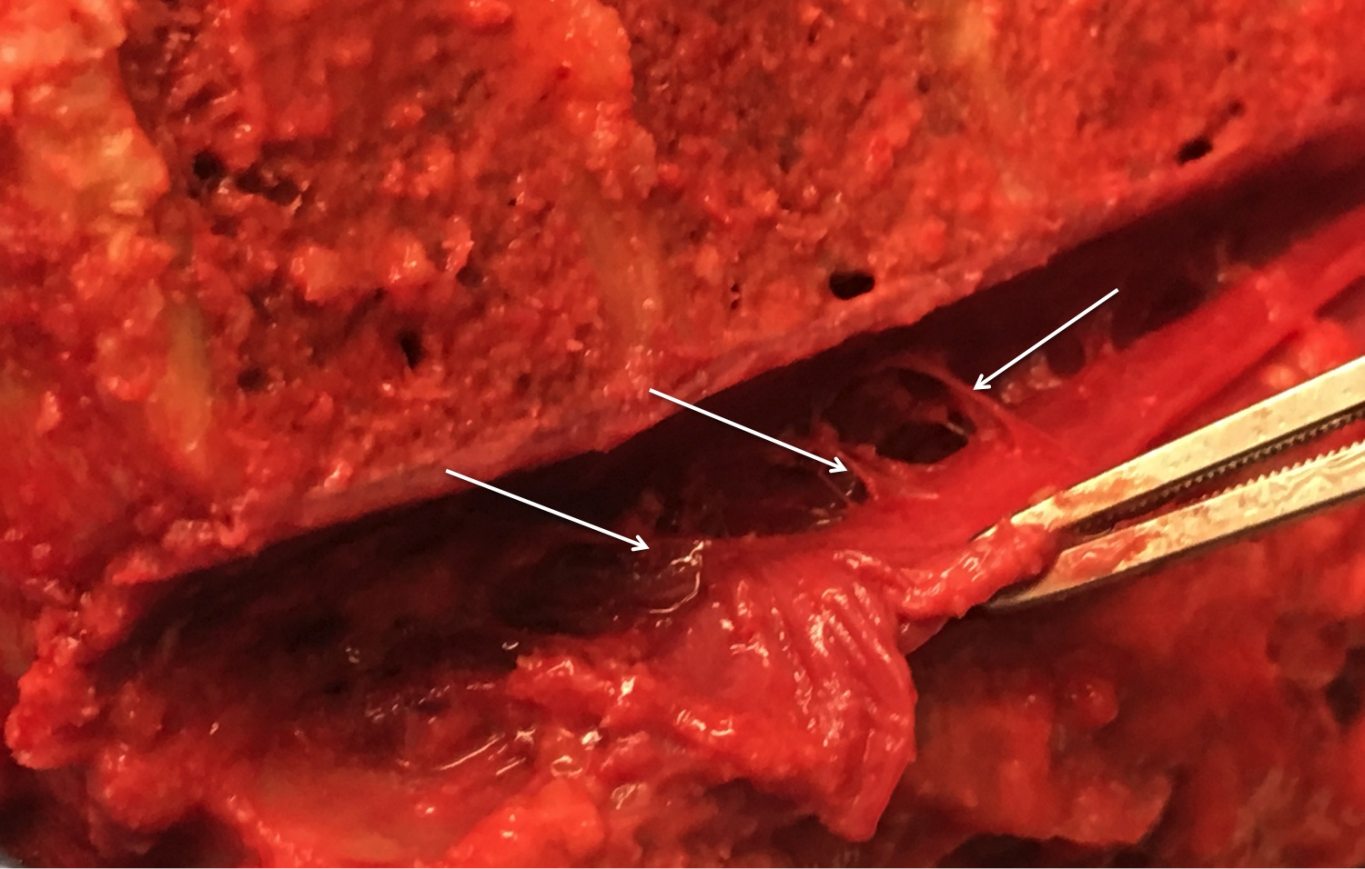
Fresh cadaveric dissections noting Hofmann’s ligaments (arrows) here attaching anteriorly to the PLL.

**Figure 3:**
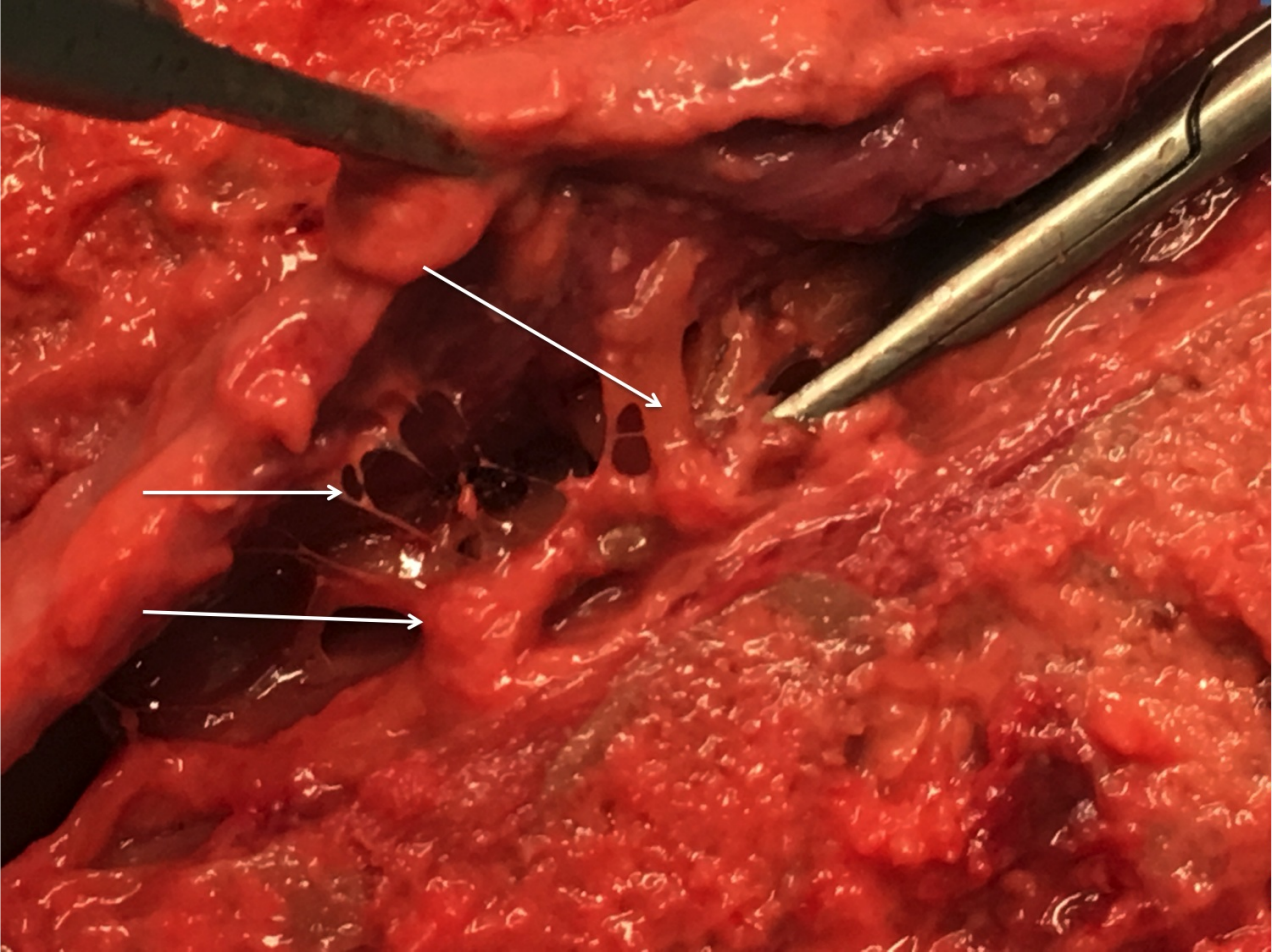
Fresh cadaveric specimen demonstrating varying sizes of Hofmann’s ligaments (arrows).

Hofmann’s ligaments have been described as fibrous connective tissue bands that run ventrolaterally from the dura mater to the vertebral canal [6]. Trolard (1893) had reported these ligaments earlier and restricted them to the lower lumbar spine and upper sacral canal [3,7-8]. However, in 1898, Max Hofmann defined these bands in detail [9-10]. According to Wiltse, et al. (1993) [10] and Wiltse (2000) [1], Hofmann’s ligaments have been seen to be “narrow, almost threadlike” connections between the dural sac and the PLL, being present at most levels (Figure 2 and Figure 3).

**Figure 4:**
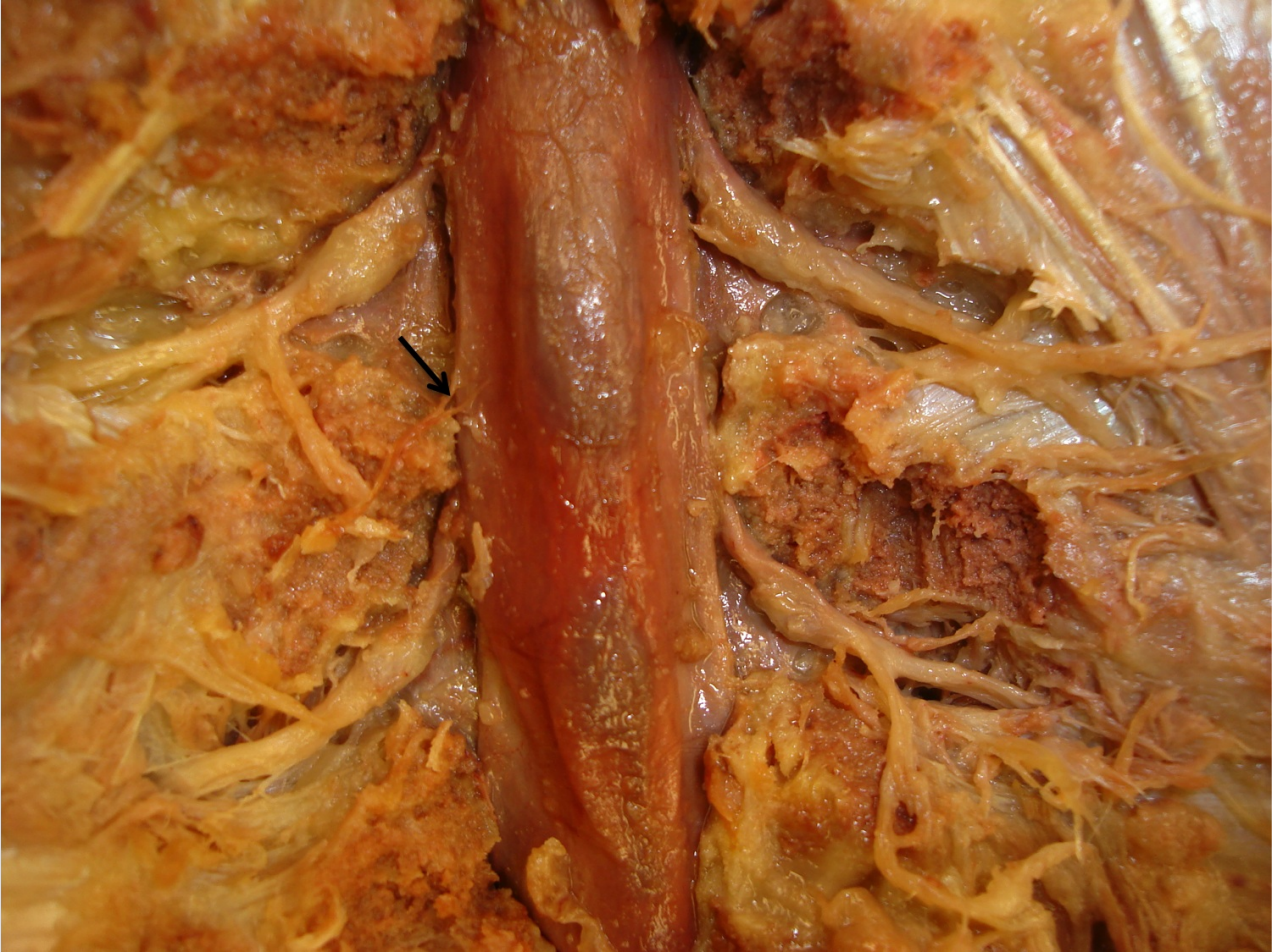
Fixed cadaveric specimen (dorsal view) noting a small lateral Hofmann’s ligament (arrow).

These authors also found that these connections might be more complex and varied than Hofmann originally described, having three or four fibrous connections per level [1,10]. Contrary to Wiltse, et al. (1993) [10], some authors have found that the quantity of ligaments differs at various vertebral levels and between species [9]. There were three sets of Hofmann’s ligaments based on their connections; 1) midline (from anterior dural sac to PLL), 2) lateral (from anterolateral dura to the lateral extent of the PLL) and 3) proximal root sleeve (from the dural extension of the nerve root sleeve to the PLL and periosteum of the inferior pedicle [11]. True Hofmann’s ligaments included only the midline and lateral ligaments as the proximal root sleeve attachments were not acknowledged by Hofmann [10]. In previous studies, Hofmann’s ligaments were seen to be “thick and well developed” in the lower lumbar vertebrae, at times broadening into lengthwise sheets that were placed over an extensive range in the lengthwise level, and at upper lumbar regions the ligaments were observed to be less well developed and the attachments “thin and scanty” [11]. Hofmann’s ligaments above the L1 vertebrae seemed to have never been noted [12]. In a study by Wadhwani, et al. [11], Hofmann’s ligaments were seen in all cadavers studied (n=18) between C7 and L5 varying in number and length at each level (Figure 4).

The majority of the ligaments were confined to an individual vertebral segment (1-5 ligaments) but some extended over many segments. Hofmann’s ligaments were found to attach both medially and laterally to the PLL, also varying in its connections with the dural sac, but all of Hofmann’s ligaments attached to the anterior surface of the dural sac. According to Wadhwani, et al. (2004) [11], the PLL was loosely attached to the middle of the dura mater along the vertebral column but the ventral dural sac was observed to be adhered intimately to the PLL at different levels of the cervical and thoracic vertebrae in different cadavers (C6, C7, T1 and T2).The orientation of Hofmann’s ligaments was seen to be consistent, running in a caudocranial fashion from the dura to the PLL at cervical and upper thoracic levels while in the lumbar vertebrae, a craniocaudal orientation was present. The ligaments at T8 to T9 lay almost at right angles between the dural sac and the PLL and became more oblique nearing the ends of the spine. This arrangement of these ligaments suggests a supportive and protective role in stabilizing and anchoring the dural sac and by that, the spinal cord and spinal nerves, to the bony vertebral canal. The greatest number of ligaments were observed in the lower thoracic spine. Yong-Hing, et al. [13], linked the difference between the structure of the connective tissues of the posterior vertebral column versus the anterior dural ligaments with a potential difference in function. Yong-Hing, et al. [13] emphasized that a function of mobility required more elasticity, such as the ligamentum flavum, in contrast to the function of stabilization and immobility, which required a more fibrous nature to the connective tissue. The connection of the anterior dura mater to the PLL by Hofmann’s ligaments produces further support for the dural sac, with Hofmann’s ligaments securing it near to the posterior surface of the vertebral bodies and intervertebral discs. The presence and function of Hofmann’s ligaments are in agreement with the statement that the dural sac does not collapse after death, even when there is no longer any support from the cerebrospinal fluid pressure, which, more or less, does not exist in the cadaver [14].

### Embryology

Studies have shown that Hofmann’s ligaments were first seen at 39 weeks’ gestation but there was no literature on their formation [10,15]. Hamid, et al. (2002) [15] in the morphological and developmental study of the human lumbar anterior epidural space reported that the adult and the 39-week fetus showed the greatest similarities. Munkácsi (1990) [16] dissected 12 fetuses and found Hofmann’s ligaments in specimens of 50 mm CR length. This is similar to the findings of Hamid, et al. (2002) [15] where the epidural space became filled with connective tissue at 13 weeks gestational age. Wadhwani, et al. [11] stated that the loose fibrous attachments between the anterior dural sac and the PLL more or less stay together during fetal growth. However, due to more movement of the spinal column after birth , this loose tissue rearranges into a set of fibrous bands (ligaments). This is congruent with the statement of Hamid, et al. (2002) [15] that Hofmann’s ligaments are present at birth. During initial growth, the PLL is closely adhered to the anterior dura mater. Munkácsi, et al. [16] described two types of epidural ligaments, one formed between the dural sac and the lateral border of the PLL and the second type between the anterior dura and the PLL.

### Proposed functions

One proposed function of Hofmann’s ligaments, early in development, is to keep the dura against the vertebrae as the spine lengthens [11]. When the intervertebral disc places pressure on the anterior dural sac, Hofmann’s ligaments may also play a protective role in limiting movement of the spinal nerves posteriorly preventing stretching of the spinal nerve roots [1], and thereby pain [15]. On the contrary, Munkácsi [16] reported clinical [17-20] and anatomical [9] studies, which indicated that the epidural ligaments contributed to the pathogenesis of nerve root compression in the sciatica syndrome, originally [21] caused by herniated discs in the vertebral canal. Wiltse [1] stated that with the prevention of movement of the spinal nerves, pain is produced due to the pressure anteriorly, although there is sufficient space posteriorly for the nerve in the bony canal. Wadhwani, et al. [11] and Spencer, et al. (1983) [9] posited that Hofmann’s ligaments may contribute to the pathogenesis of sciatica due to stress on an attached nerve root, as well as cause somatic pain by pulling on the PLL. This theory is supported by the fact that the degree of the protruded intervertebral disc and the severity of the neurological symptoms do not always match [9,16-17,19-20,22].

### Clinical application

There is no mention in the literature of how the epidural ligaments, particularly the posterior ligaments in the lumbar region, might affect the distribution and thus, the efficacy of epidural anesthetic blockade. Prior studies have shown that the posterior epidural ligaments divide the posterior/dorsal epidural space into anterior and posterior compartments [6]. However, other studies on spinal flexion and extension [23] have determined that the dural sac is allowed greater displacement posteriorly as opposed to anteriorly. This is in accordance with the findings of Parkin and Harrison (1985) [14] who observed the anterior epidural ligaments to be “firmer and shorter” than the posterior connections. Thus, the adequacy of posterior epidural anesthesia may not be affected by the anatomical arrangement of the dorsal epidural ligaments.

Lastly, does the anatomical topography of the epidural ligaments contribute to spinal posterior epidural abscesses being more common than anterior epidural abscesses? There have been no studies that have found the answer to this question. Likewise, it is unknown if the location of spinal epidural abscesses is affected by the anatomy and location of epidural ligaments. Spinal epidural abscesses have an incidence of about 0.2 to 2.8 cases per 10,000 hospital admissions [24-38] and are primarily located in the thoracic and lumbar regions but can occur in the cervical and sacral spine as well [25,31,34-35,37,39-45]. Studies have reported that spinal epidural abscesses are more commonly found in the dorsal epidural space followed by the ventral epidural space and lastly, circumferentially [34,41]. It is possible that at least early loculation of such abscesses could be due to or contributed to by Hofmann’s ligaments.

## Conclusions

This comprehensive review of Hofmann’s ligaments expands on our understanding of their anatomy and function, increasing the awareness of their presence and serves as an impetus for further study of their histology, innervation and function. These ligaments are clearly a normal anatomical finding and should be further elucidated to better understand their role in movement and displacement of the dura mater within the spinal canal and its relationship to pain and the spread of epidural anesthesia.
